# Study on Optimal Selection of Wavelet Vanishing Moments for ECG Denoising

**DOI:** 10.1038/s41598-017-04837-9

**Published:** 2017-07-04

**Authors:** Ziran Peng, Guojun Wang

**Affiliations:** 1 School of Information Science and Engineering, Central South University, Changsha, 410083 China; 2 School of Computer Science and Educational Software, Guangzhou University, Guangzhou, 510006 China; 3 Hunan Vocational College of Commerce, Changsha, 410205 China

## Abstract

The frequency characteristics of wavelets and the vanishing moments of wavelet filters are both important parameters of wavelets. Clarifying the relationship between the wavelet frequency characteristics and the vanishing moments of the wavelet filter can provide a theoretical basis for selecting the best wavelet. In this paper, the frequency characteristics of wavelets were analyzed by mathematical modeling, the mathematical relationship between wavelet frequency characteristics and vanishing moments was clarified, the optimal wavelet base function was selected hierarchically according to the amplitude frequency characteristics of ECG signal, and an accurate notch filter was realized according to the frequency characteristics of the noise. The experimental results showed that the optimal orthogonal wavelet analysis for the ECG signals with different frequency characteristics could make the high frequency energy distribution sparser, and the method proposed in this paper could effectively preserve the singularity of the signal and reduce the signal distortion.

## Introduction

Electrocardiogram (ECG) signals collected from the surface of the human body inevitably contain noise, such as from EMG interference and power frequency interference. Removal of this noise is required for further analysis of ECG signals. At present, digital filtering algorithms used with ECG mainly include EMD decomposition, wavelet transform, and other methods. In ref. 1, Sharmila and P. Geethanjali proposed an adaptive ECG signal denoising method^2^. Ensemble empirical mode decomposition (EEMD) and a threshold-based genetic algorithm (GA) are used to remove mixed noise by using a similarity measurement method based on a probability density function. The experimental results in this paper show that this method has certain advantages, however, adaptive threshold estimates are based on probability and statistics and, therefore, contain not only the complexity of the calculation but also the uncertainty of the results. Reference 3 proposes an IMF threshold technique based on GAs. The experimental results show that this method is superior to the traditional method based on EMD noise reduction. In ref. 4, an ECG signal enhancement technique is proposed for the elimination of noise components. By eliminating ECG signal noise in the time domain, the signal-to-noise ratio (SNR) can be improved while maintaining a lower RMSE (root mean square error). The methods discussed in refs 3 and 4 do not consider the characteristics of the signal itself, so it is possible to eliminate singular points in the signal while denoising. The EMD-based algorithm has some limitations, and its practical application is limited. At present, wavelet transform is widely used in biomedical engineering. Reference 5 proposes a method for extraction of ECG signals based on wavelet analysis. Firstly, CWT (continuous wavelet transform) is performed on signals obtained from human abdomen and chest regions, and the decomposition coefficient is processed by an LMS algorithm. The correlation of the processed wavelet coefficient is then calculated. Experimental results show that this method is superior to the EMD algorithm in noise immunity. However, there is no further discussion on how to choose the wavelet basis function and how to deal with it hierarchically. In recent literature, the main methods of dealing with physiologically-based digital signals are the classical wavelet transform and lifting format wavelet transform. However, most of the methods using wavelet transform involve specific parameters and features of wavelets, e.g., wavelet vanishing moments, wavelet support length, etc. The types of wavelet basis used, as well as other parameters, are mainly determined by experience and experiment, and there is a lack of systematic theoretical analysis. Based on the analysis of vanishing moment and frequency characteristics of the wavelet filter, this paper analyze how to select and use the appropriate wavelet basis function and how to filter the noise of the specific frequency characteristic in the process of wavelet transform^6^.

## Wavelet vanishing moments and optimal wavelet basis selection

For ECG signal analysis and processing by wavelet transform, there are several purposes: denoising, feature detection, and data compression. Whatever the purpose, it is hoped that, after the wavelet decomposition of the ECG signal, the signal energy can achieve the maximum concentration in low-pass components, and the energy of high-pass components can achieve maximum thinning^7^. According to the current literature, under the premise of not considering the computational complexity of wavelet transform, it is generally thought that the higher vanishing moment of the wavelet can produce a better effect^8^. Some other articles contend that the effect of high-pass component thinning and the shape of the wavelet scale function are related to the similarity of the shape of the signal^9, 10^. This paper provides an in-depth study on this issue and offers an answer.

### Wavelet vanishing moment and filter amplitude-frequency characteristics

For any N, there are 2N non-zero real-scale coefficients ${h}_{0},{h}_{1}\cdots {h}_{2N-1}$, which can constitute a [0, 2N−1] scale function and wavelet function. With an appropriate choice of these coefficients, the 2N−1 order polynomial can be written as: ${H}_{N}(z)={(z+1)}^{N}Q(z)$, where Q(z) is the N−1 order polynomial, and *Q*(−1) ≠ 0. *H*_*N*_(*z*) is the N-order vanishing moment, and is subject to Taylor expansion at z = −1:

1 $$\begin{array}{rcl}{H}_{N}(z) & = & {H}_{N}({\rm{1}})+{H}_{N}^{^{\prime} }({\rm{1}})(z+1)+\frac{{H}_{N}^{^{\prime\prime} }({\rm{1}})}{{\rm{2}}!}{(z+1)}^{2}+\cdots \\  &  & +\frac{{H}_{N}^{(N)}({\rm{1}})}{{\rm{N}}!}{(z+1)}^{N}+R(N+1)\end{array}$$

At *i* < *N*, ${H}_{N}^{(i)}({\rm{1}})=0$, the above formula can be expressed as:

2 $${H}_{N}(z)\approx \frac{{H}_{N}^{(N)}({\rm{1}})}{{\rm{N}}!}{(z-1)}^{N}$$

The Fourier transform is a special case of the Laplace transform in imaginary axis *s* = *j*Ω, and thus is mapped as a unit circle in the Z-plane. The Z-transform of the sequence H on the unit circle is equal to the ideal Fourier transform of the sampled signal^10, 11^. Let the digital frequency (ω) be the parameter of the unit circle in the Z-plane. Ω is the angle of the Z-plane, denoted as ${A}_{0}=\frac{{H}_{N}^{(N)}({\rm{1}})}{{\rm{N}}!}$. Then,

3 $$H(\omega )\approx {A}_{0}{({e}^{jw}+1)}^{N}={{\rm{2A}}}_{0}{{\rm{e}}}^{\frac{jNw}{2}}{(\cos \frac{w}{2})}^{N}$$

For the orthogonal wavelet, the perfect reconstruction satisfies:

4 $$\begin{array}{c}H(z)H({z}^{-1})+{\rm{H}}(-z){\rm{H}}(-{{\rm{z}}}^{-1})={\rm{2}}\\ H(z)G({z}^{-1})+{\rm{H}}(-{\rm{z}}){\rm{G}}(-{{\rm{z}}}^{-1})={\rm{0}}\end{array}\}$$

Therefore: $$G(z)=-{z}^{-1}H(-{z}^{-1})=-{z}^{-1}{A}_{0}{(-{z}^{-1}+1)}^{N}\circ $$

5 $${\rm{Namely}}:\quad \quad \quad \quad \begin{array}{rcl}G(\omega ) & \approx  & -{A}_{0}{e}^{jw}{(-{e}^{-jw}+1)}^{N}=-{A}_{0}{e}^{jw}{({e}^{-jw+\pi }+1)}^{N}\\  & = & {\rm{2}}{A}_{0}{e}^{-\frac{jNw}{2}+\frac{N\pi }{2}}{(\sin \frac{w}{2})}^{N}\end{array}$$

#### **Theorem 1**

: The order of the vanishing moment of an orthogonal wavelet is proportional to its corresponding filter order.

#### **Proof**

: For $H(\omega )\approx {{\rm{2A}}}_{0}{{\rm{e}}}^{\frac{jN\omega }{2}}{(\cos \frac{\omega }{2})}^{N}$, take the first derivative, resulting in the rate of change with $\mathop{\mathrm{lim}}\limits_{w\to 0}\frac{{\rm{\Delta }}H(\omega )}{{\rm{\Delta }}w}$:

6 $$\frac{dH(\omega )}{d\omega }\approx -N{{\rm{A}}}_{1}{(\sin \frac{\omega }{2})}^{N-1}$$

Similarly,

7 $$\frac{dG(\omega )}{d\omega }\approx {\rm{N}}{{\rm{.A}}}_{1}{(\cos \frac{\omega }{2})}^{N-1}$$

**Figure 1:**
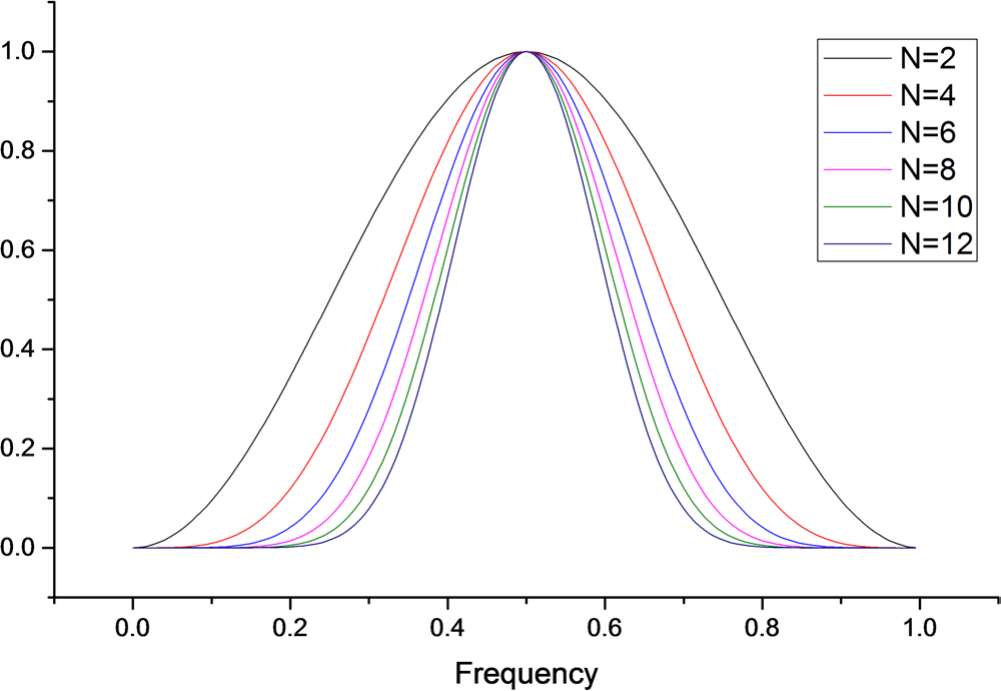
Amplitude-frequency characteristics of the orthogonal wavelet high pass filter with vanishing moments N.

In the transition band $(\frac{4\pi }{16}\le \omega  < \frac{5\pi }{16})$ of the high-pass filter, the approximation of ${(\cos \frac{\omega }{2})}^{N-1}$ is equal to 1, so the filter slope at the edge of the transition band is: $\frac{dG(\omega )}{d\omega }\approx N{{\rm{A}}}_{1}$. This shows that the larger the vanishing moment, the steeper the slope of the edge in the transition band of the corresponding filter, and the higher the order of the filter Figure 1 shows the amplitude-frequency characteristics of orthogonal wavelet high-pass filters with vanishing moments of 2, 4, 6, 8, 10, and 12.

### Selection of wavelet for ECG signal processing

It can be deduced from the above cases that the low and high frequency energies after wavelet transform are related to the frequency characteristics of the signal and also to the vanishing moments of the wavelet. For a signal whose frequency is stable, there must be a wavelet with a vanishing moment of N, so that the high-frequency energy after the wavelet transformation is the lowest^12, 13^. Therefore, the following theorem can be introduced:

#### **Theorem 2**

: The signal *f*(*x*) is a periodic signal with stable frequency characteristics. For a wavelet series W with order vanishing moments from N1 to N2, there exists a unique wavelet W_k_ (vanishing moment k: N1kN2) such that its energy is minimized after wavelet transform.

#### **Proof**

**:** According to the Mallat principle, let *x*_*j*_(*k*), *d*_*j*_(*k*) be a discrete approximation coefficient in multiresolution analysis where *h*_0_(*k*), *h*_1_(*k*) are two filters that satisfy the orthonormal two-scale difference equation. Then, for *X*_*j*_(*k*), *d*_*j*_(*k*), there are recursive relations as follows:

8 $$\begin{array}{c}{x}_{j+1}(k)=\sum _{n=-\infty }^{\infty }{x}_{j}(n){h}_{0}(n-2k)={x}_{j}(k)\ast {\bar{h}}_{0}(2k)\\ {d}_{j+1}(k)=\sum _{n=-\infty }^{\infty }{x}_{j}(n){h}_{1}(n-2k)={x}_{j}(k)\ast {\bar{h}}_{1}(2k)\end{array}\}$$

If $X(z)=Z[x(n)],H(z)=Z[h(n)]$, then, in accordance with the Fourier convolution theorem:

9 $${x}_{j+1}(k)={x}_{j}(k)\ast {\bar{h}}_{0}(2k)=X(z)H({z}^{2})$$

Also, according to the Parseval theorem, the following equation exists:

10 $$\sum _{n=-\infty }^{\infty }x(n){h}^{\ast }(n)=\frac{1}{2\pi j}\mathop{\sqint}\limits_{C}X(\upsilon )H(\upsilon ){\upsilon }^{-1}d\upsilon $$

At *x*(*n*) = *h*(*n*),

11 $${\sum _{n=-\infty }^{+\infty }|x(n)|}^{2}=\frac{1}{2\pi }{{\int }_{-\pi }^{+\pi }|X(j\omega )|}^{2}d\omega $$

Therefore,

12 $${\sum _{k=-\infty }^{+\infty }|{x}_{j+1}(k)|}^{2}=\frac{1}{2\pi }{\int }_{-\pi }^{+\pi }{|X(j\omega )H(2j\omega )|}^{2}d\omega $$

At $$\frac{9\pi }{16}\le \omega  < \pi $$,

13 $${{\int }_{\frac{9\pi }{16}}^{+\pi }|X(j\omega )H(2j\omega )|}^{2}d\omega ={{\int }_{\frac{9\pi }{16}}^{+\pi }|{A}_{0}X(j\omega )|}^{2}d\omega ={M}_{0}$$

At $\omega  < \frac{\pi }{2}$, *H(ω*) = 0; at $\frac{\pi }{2}\omega  < \frac{9\pi }{16}$, $H(\omega )\approx n{A}_{0}(\omega -{\omega }_{0})+{B}_{0}$; therefore,

14 $$\frac{1}{2\pi }{{\int }_{-\pi }^{+\pi }|X(j\omega )H(j2\omega )|}^{2}d\omega =\frac{{M}_{0}}{2\pi }+\frac{1}{2\pi }\sum _{kD=\frac{\pi }{2}}^{\frac{9\pi }{16}}{|X(jkD)[N{A}_{0}(2kDj-{\omega }_{0})+{B}_{0}]|}^{2}$$

Let

$f(n)={\sum }_{kD=\frac{\pi }{2}}^{\frac{9\pi }{16}}{|X(jkD)[n{A}_{0}(2kDj-{\omega }_{0})+{B}_{0}]|}^{2}$, ${a}_{j}=X(j{k}_{j}D),{\omega }_{j}={A}_{0}(2kDj-{\omega }_{0})$; with the necessary conditions for extreme values with the function, we get,

15 $$\frac{df}{dn}=2\sum _{j=1}^{N}{a}_{j}[n{\omega }_{j}+{B}_{0}]{\omega }_{j}=0$$

Obviously, at ${\sum }_{j=1}^{N}{a}_{j}{\omega }_{j}\ne 0$, there is a sole linear solution:

16 $$n=\lfloor \frac{{\sum }_{j=1}^{N}{a}_{j}{\omega }_{j}}{{\sum }_{j=1}^{N}{a}_{j}{B}_{0}}\rfloor $$

**Figure 2:**
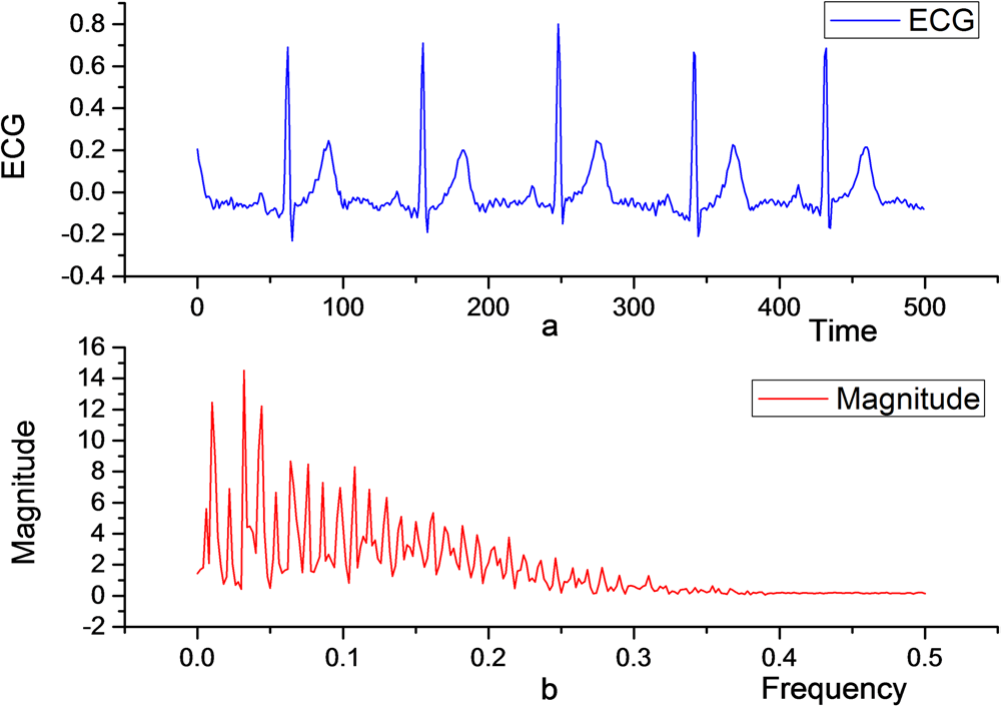
Real-time ECG image (**a**), and its frequency characteristics (**b**).

In general, for the signal processing, there are minimum requirements on the order of the vanishing moments in the filter^14^. The reason being that, the lower the vanishing moments, the more signal detail is lost during processing and the greater the distortion. If the lower limit of the vanishing moments is given, it can be understood from Theorem 2 that for a given a signal and set of wavelets with given frequency characteristics, there must be an optimal wavelet for minimizing the energy contained in the processed high-pass component. This facilitates the subsequent filtering process and the signal compression process. Real-time ECG signal Fourier transform is as shown in Fig. 2a, and we can clearly find the corresponding frequency characteristics, as shown in Fig. 2b.

**Figure 3:**
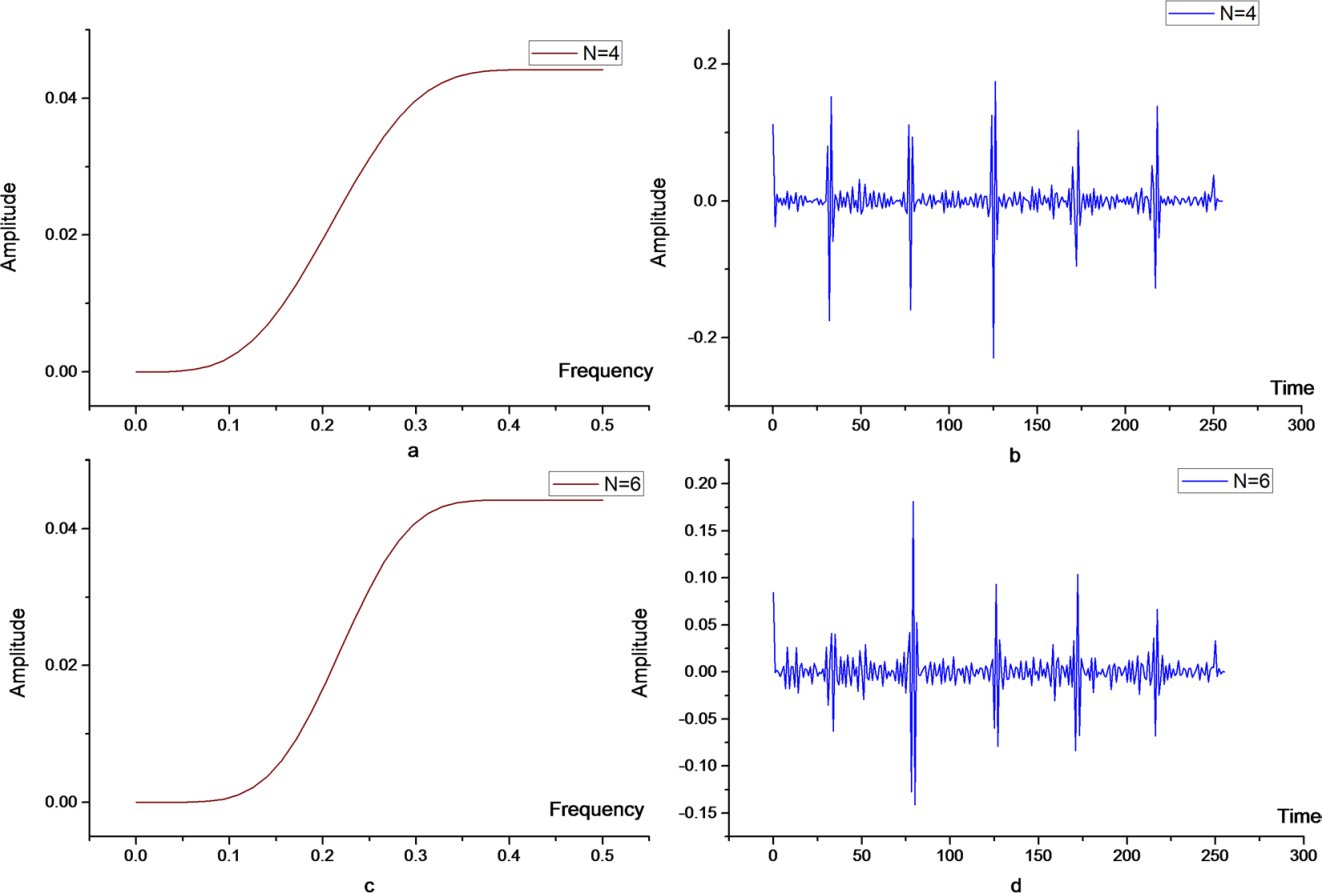
High frequency components of ECG signals after 4- (above) and 6-order vanishing moments (below).

**Figure 4:**
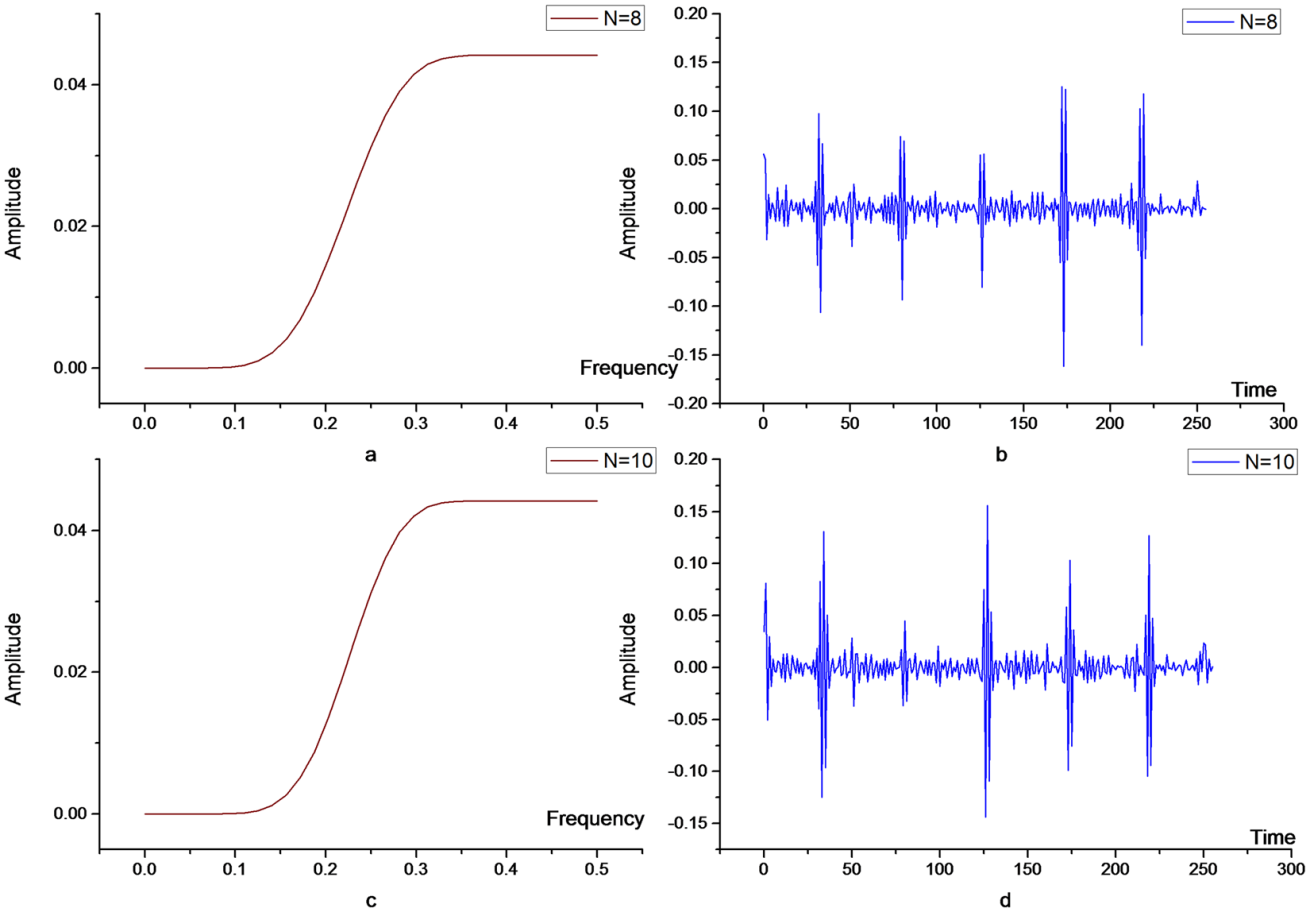
High frequency components of ECG signals after 8- (above) and 10-order vanishing moments (below).

The real-time ECG signals are decomposed by four-order, six-order, eight-order and ten-order orthogonal filters, respectively, and the corresponding high-pass components are shown in Figs 3b,d and 4b,d. As shown in Table 1, the energy of the decomposed high-pass signal is smallest when the wavelet vanishing moment N = 6. It can be seen that the orthogonal wavelet with the vanishing moment N = 6 is the best choice when the first layer is decomposed.

**Table 1:** Total energy of the high frequency system after wavelet DBN decomposition.

DBN	DB4	DB6	DB8	DB10
$E=\sum {d}_{i}^{2}$	0.316127	0.171010	0.203272	0.227824

According to Theorem 2, prior to the processing of the ECG signal, an optimal wavelet should be selected to achieve filtering. There should be a complete wavelet set, where the wavelet should have a lowest vanishing moment such as four-order, until the fast wavelet transform can support up to the highest order, such as 24-order. If the vanishing moment is too low, then it will affect the accuracy of signal processing; if the vanishing moment is too high, the calculation will be too complex^15^. The high-frequency filter bank of each wavelet is extended by zero-padding (length Γ), and then Fourier-transformed. The typical 2-cycle ECG signal (length Γ) is taken as a sample, and is Fourier-transformed. According to Formula (12), the total energy of the high-pass component corresponding to the vanishing moment wavelet is obtained, and then the wavelet with the smallest energy is selected as the optimal choice.

## Hierarchical processing in ECG signal wavelet transform

### A filter with a different vanishing moment is selected according to the frequency characteristics of the signal in each layer

**Figure 5:**
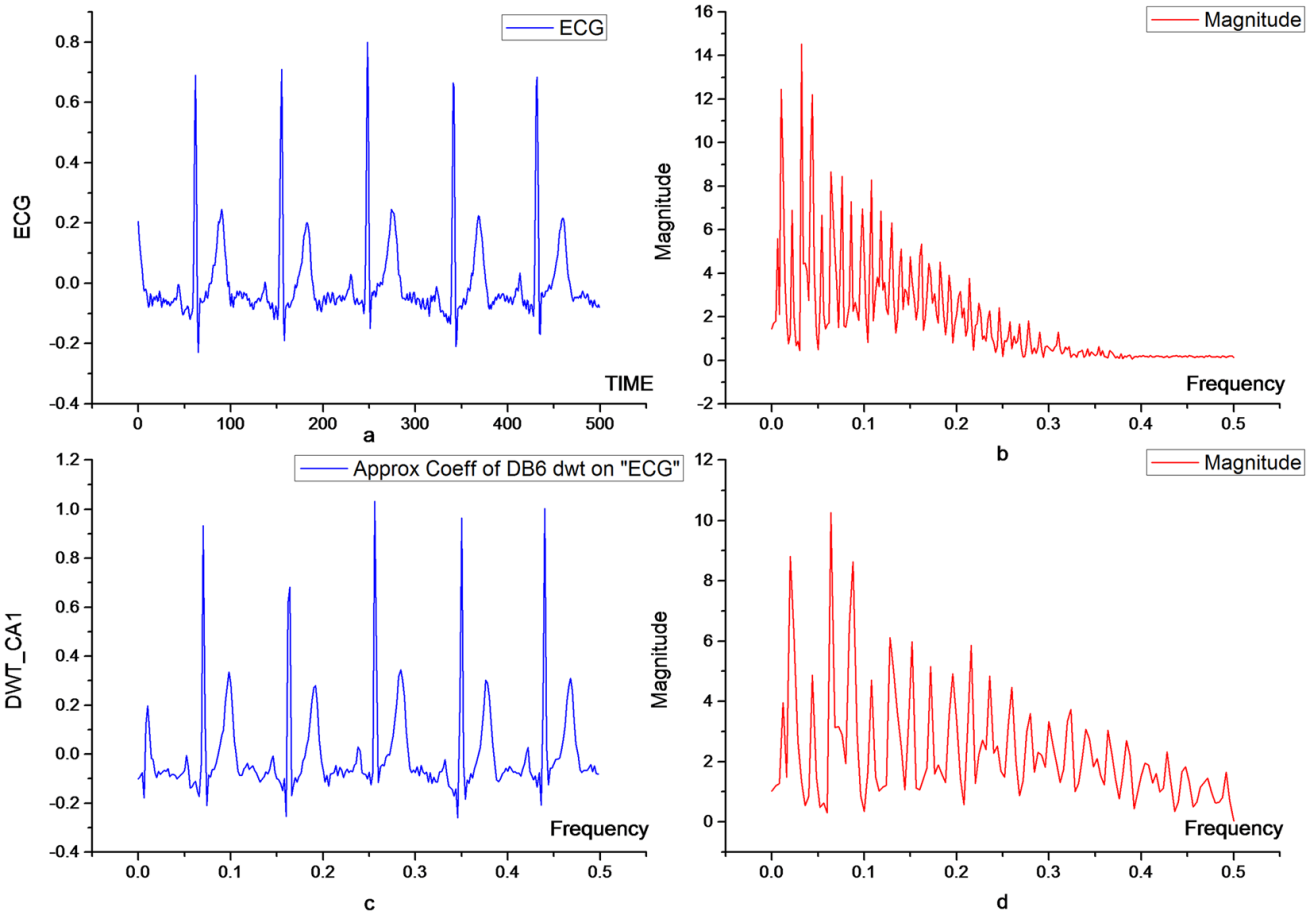
Low frequency component and frequency characteristics of ECG signal after DB6 filter.

After a layer of wavelet is decomposed, low-pass components enter a new layer of wavelet transform. In this wavelet decomposition, should the original filter or the more matched filter for processing be used? It is clear that the sampling frequency is reduced by a factor of two, and so is the frequency of the signal after the bisectional extraction. However, since the processed signal contains only the low-pass portion of the original signal, there is a significant difference in the spectral distribution between layers j + 1 and j. In order to let the high-frequency components contain less energy, we should reselect an optimally matched filter for processing. Figure 5b shows the frequency characteristics of the original signal. Figure 5d shows frequency characteristics of low-frequency signals after wavelet decomposition.

**Figure 6:**
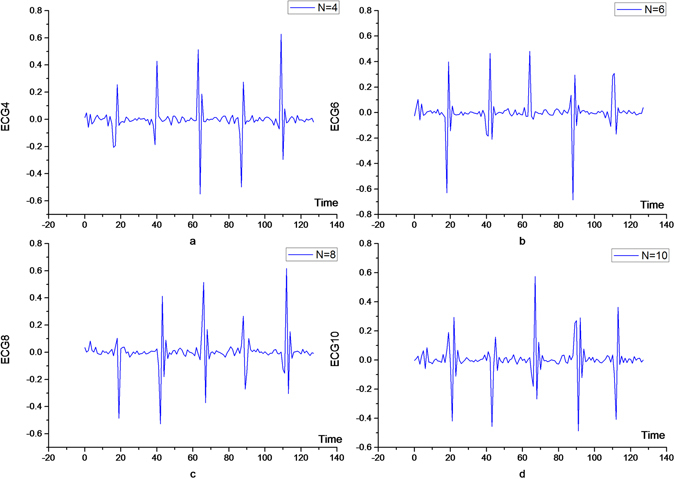
High frequency component of the ECG signal through the N-order vanishing moment filter.

As can be seen from Fig. 5, there is a clear difference between the original and filtered frequency distributions. In this paper, the decomposed low-pass signals are re-decomposed through the filter with vanishing moments of 4, 6, 8, and 10. The high-pass components after such decomposition are shown in Fig. 6a,b,c and d, respectively. It can be seen that there is a significant difference. At N = 4, the total energy of the decomposed high-pass component is the smallest (the specific data are shown in Table 2). This indicates that, in the wavelet transform process, it is necessary to choose wavelets with different vanishing moments to deal with the different levels of wavelet transform, so as to realize the optimization of energy distribution. In general, for the first and second layers, different filter banks should be used and the rest of the layers can be determined according to need.

**Table 2:** Total energy of the high frequency coefficients after wavelet DBN decomposition.

DBN	DB4	DB6	DB8	DB10
$E=\sum {d}_{i}^{2}$	1.841969	2.00026	1.965258	1.862453

### Wavelet transform to remove power frequency interference

One of the main sources of noise is 50 Hz frequency interference, which is generally removed prior to further signal analysis and processing. If using Fourier-transform for the mathematical signal processing, this problem can easily be addressed by setting the corresponding frequency component to 0. However, the problem of how to use wavelet transform for frequency notching has not been discussed in the literature. The general practice is to set the threshold for high-frequency components for filtering, but the desired accuracy of the results is difficult to achieve^16, 17^.

If the wavelet transform is made for signal f (x) with sampling frequency P0, then the high-pass component will contain the frequency component from $[\frac{{P}_{0}}{4},\frac{3{P}_{0}}{4}]$ and the low-pass component will contain frequency components: $[0,\frac{{P}_{0}}{4}]\cup [\frac{3{P}_{0}}{4},{P}_{0}]$ between high-pass filter $G(\omega )\approx {\rm{2}}{A}_{0}{e}^{-\frac{jNw}{2}+\frac{N\pi }{2}}{(\sin \frac{w}{2})}^{N}$ and low-pass filter $H(\omega )\approx -{{\rm{2A}}}_{0}{{\rm{e}}}^{\frac{jNw}{2}}{(\cos \frac{w}{2})}^{N}$, there are two intersection points in the region [0, 2*π*]: $(\frac{\pi }{4},G(\frac{\pi }{4})),(\frac{5\pi }{4},G(\frac{5\pi }{4}))$. These two points are also a transition area from low frequency to high frequency. From the Fourier convolution theorem, we can see that the effect of *G*(*ω*), *H*(*ω*) on the signal is equivalent to the transfer function in the analysis of the filter circuit. In the following, we further analyze the suppression factor of the signal at these two critical points:

$G(\frac{\pi }{4})\approx \Vert {\rm{2}}{A}_{0}{e}^{-N(\frac{j\pi }{8}-\frac{\pi }{2})}{(\sin \frac{\pi }{8})}^{N}\Vert$ = ${\rm{2}}{A}_{0}{(\sin \frac{\pi }{8})}^{N}$ = ${\rm{2}}{A}_{0}{(\frac{\sqrt{2-\sqrt{2}}}{2})}^{N}$; For ${A}_{0}=\frac{{H}_{N}^{(N)}({\rm{1}})}{{\rm{N}}!},{|H(z)|}^{2}+$ ${|H(-z)|}^{2}={\rm{1}}$; so $H(z)\le {|H(z)|}^{2}\le {\rm{1}}$. Apparently, ${A}_{0}\le \frac{{\rm{1}}}{{\rm{N}}!}$. Therefore: $G(\frac{\pi }{4})\approx {\rm{2}}{A}_{0}{(\frac{\sqrt{2-\sqrt{2}}}{2})}^{N}\le \frac{{\rm{2}}}{{\rm{N}}!}{(\frac{\sqrt{2-\sqrt{2}}}{2})}^{N}$. Obviously, this is a function of N featuring faster convergence. For example, at N = 4, $G(\frac{\pi }{4})\approx {\rm{0.00198178}}$; at N = 6, $G(\frac{\pi }{4})\approx {\rm{0.0000101872}}$. For any given positive *ε* close to 0, N_0_ is always present, such that:

17 $$\mathop{\mathrm{lim}}\limits_{{\rm{N}}\to {{\rm{N}}}_{{\rm{0}}}}(\frac{{\rm{2}}}{{\rm{N}}!}{(\frac{\sqrt{2-\sqrt{2}}}{2})}^{N}-\varepsilon )=0.$$

So, when the appropriate vanishing moment (N) is taken, it is possible to make the suppression factor of the critical point infinitely small, so that the gain of the signal outside the region of this point is close to zero, theoretically equivalent to the truncated state.

Let the sampling frequency of the signal at P0, which contains the noise at the frequency of ${P}_{s}=\frac{{P}_{0}}{2}$. Suppose that the frequency tolerated in denoising through notching is: $[{P}_{s}-\Delta P,{P}_{s}+{\rm{\Delta }}P]$, where Δ*P* is the frequency bandwidth increment. After a wavelet transform, the high-pass component contains the frequency component from $[\frac{{P}_{0}}{4},\frac{3{P}_{0}}{4}]$; and the low-pass component contains $[0,\frac{{P}_{0}}{4}]\cup [\frac{3{P}_{0}}{4},{P}_{0}]$. If this time the band of the high-pass component is wide, and if this part of the signal is removed by setting at zero, then, undoubtedly, the effective frequency will be removed; or if there is a cutoff according to the threshold, noise will not be filtered completely. Both of these conditions can cause signal distortion. It is clear that the high-frequency component can be continuously decomposed by the wavelet, and the band of the high-frequency component is further narrowed and gradually approximated to $[{P}_{s}-{\rm{\Delta }}P,{P}_{s}+{\rm{\Delta }}P]$. Obviously, the higher the order of the filter used for the notch processing, the smaller the overlapping region of high-pass and low-pass frequencies, the steeper the filter frequency curve, and the more concentrated the energy^18^.

In order to facilitate the calculation, this paper uses the normalized frequency as the unit. The normalized frequency R of the actual frequency *P*_*t*_ in the frequency interval [*P*_1_, *P*_2_] is defined as follows:

18 $$R=\frac{{P}_{t}-{P}_{1}}{{P}_{2}-{P}_{1}}$$

Any frequency range [*P*_1_, *P*_2_] can be expressed as a real number domain [0, 1] after the normalized processing. It can be known from Shannon’s Theorem that the sampling frequency should be no less than twice of the maximum frequency in analog signal spectrum. So the normalized frequency R should be in the range of [0, 0.5] when the sampling signal is directly filtered and [0, 1] for the second layer and above filtration.

#### **Theorem 3**

, Let the range of normalized frequency of Signal S is [0, *P*_*t*_], for normalized frequency ${P}_{s}\in [0,{P}_{t}]$, if ${P}_{s}\ast {2}^{N}\approx 0.5\pm {\rm{\Delta }}P,$ Then the signal of the frequency interval D can be extracted by band-pass filtering from [*P*_1_, *P*_2_] by N wavelet transform.

#### **Proof**

: Perform a wavelet transform on Signal S, S_D_ represents the transformed low-pass component, and S_H_ represents the transformed high-pass component; the following operations can be carried out as discussed above:

If $0.5-{\rm{\Delta }}P\le {P}_{s}\le 0.5+{\rm{\Delta }}P$, it indicates ${D}_{H}\subseteq D$, and returns the result *D*_*H*_, and the algorithm ends.

If *P*_*s*_ < 0.25, it indicates $D\subseteq {S}_{D}$, and needs to perform a wavelet transform on Signal S_D_. ${P}_{s}={2}^{\ast }{P}_{s},{\rm{\Delta }}P={2}^{\ast }{\rm{\Delta }}P$. Repeat the algorithm.

If $0.25 < {P}_{s} < 0.75$, it indicates $D\subseteq {S}_{H}$, and needs to perform a wavelet transform on Signal *s*_*D*_. ${P}_{s}={2}^{\ast }({P}_{s}-0.25),{\rm{\Delta }}P={2}^{\ast }{\rm{\Delta }}P$. Repeat the algorithm.

**Figure 7:**
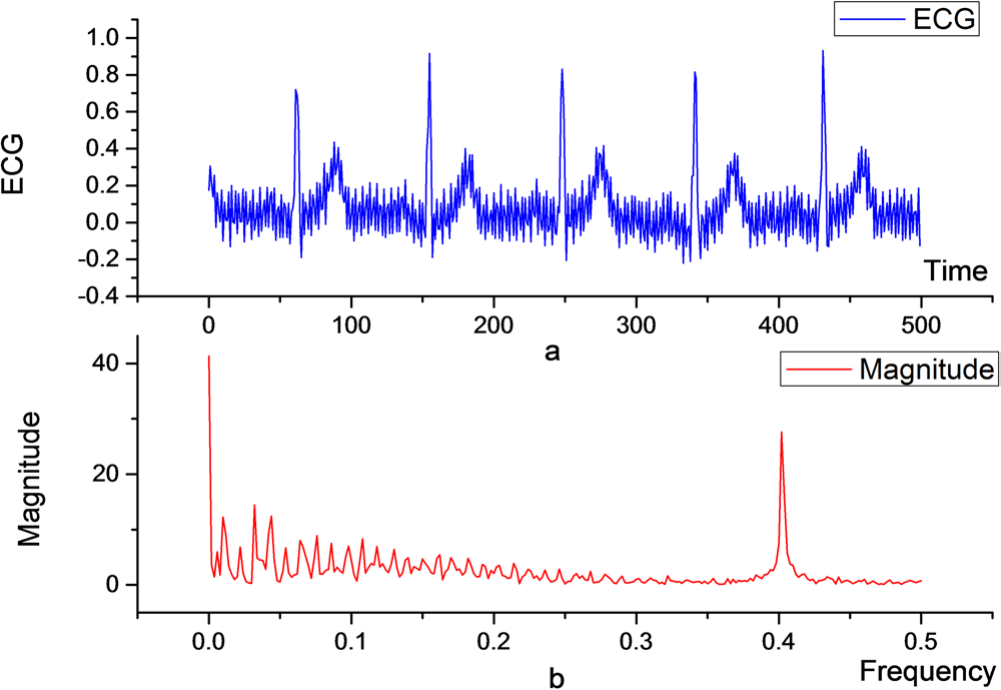
ECG signal with high frequency noise and its frequency characteristics.

**Figure 8:**
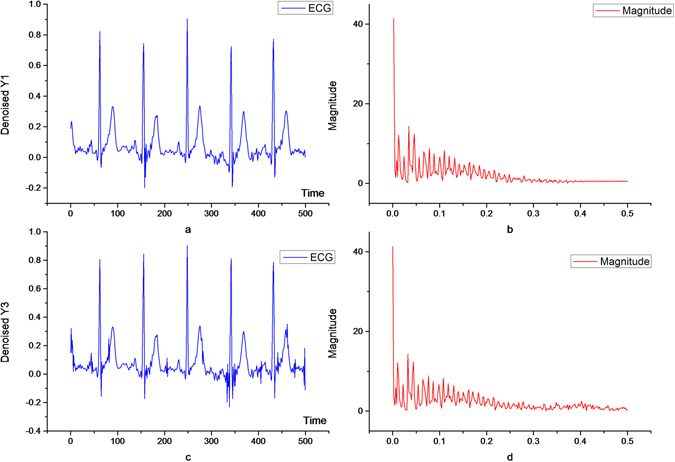
Original ECG signals filtered once (**a**,**b**) and twice (**c**,**d**) through the band-pass filter.

As shown in Fig. 7, the ECG signal has high frequency noise with a normalized frequency Freq = 0.4. Since 0.25 < Freq < 0.75, the noise is distributed in the high frequency component. The high-pass component is set to zero to complete band-pass denoising, and the effect is shown in Fig. 8a. Because the bandwidth of denoising is [0.25, 0.75], many signal details are lost. The frequency characteristics of the denoised signal are shown in Fig. 8b. The component of the signal frequency is basically zero in [0.25, 0.75].

**Figure 9:**
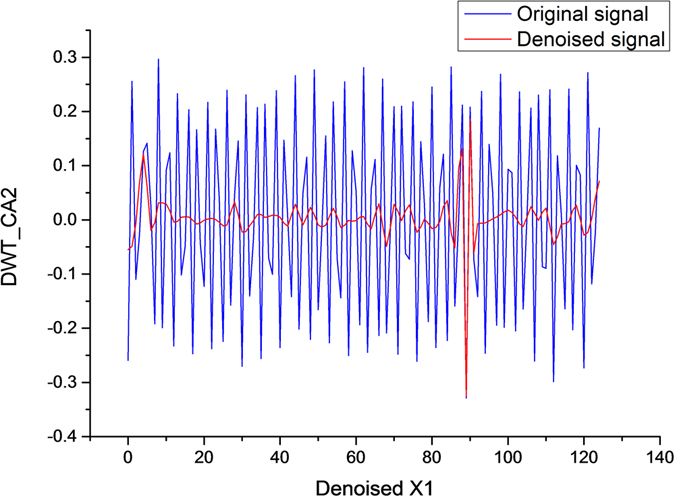
Original ECG signals twice filtered through the band-pass filter.

Therefore, there is a need for secondary filtering to implement band-pass denoising: the high-pass component after the first filtering is decomposed by the wavelet; then, the low-pass component after the secondary decomposition contains the bandwidth [0.25, 0.40] and the high-pass component contains the bandwidth [0.375, 0.500]. So, this part of the high-pass component is set to 0 to complete filtering. Afterwards, the two-part signal is recombined into the detail signal of the first layer, as shown in Fig. 9. The final filtering effect is shown in Fig. 8b. If Δ*P* = 0.100; the notching can be implemented in a very narrow area, thus preserving some of the key details of the high frequency signal.

## Experimental result

Based on the above discussion, the two key steps of the wavelet-based denoising algorithm proposed in this paper are as follows: First, the amplitude-frequency characteristic of the ECG signal is analyzed to determine the wavelet loss moment order, and on this basis, the optimal wavelet basis function is selected for different levels of wavelet decomposition. Second, by analyzing the noise frequency characteristics, determining the level and coefficient of band-pass filter, so as to realize the fixed-point removal of noise in the process of wavelet decomposition. Ten sampling records (data of the first 10 minutes for each record) are selected from ECG ID database for a contrast experiment. The adaptive genetic algorithm based on EEMD (Genetic EEMD), the adaptive threshold denoising algorithm based on discrete wavelet transform (Threshold DWT) and the wavelet denoising algorithm which is optimized by this paper (Proposed DWT) are applied for filtering processing. The filtering effect is evaluated from the aspects of filtering time consumption, denoising effect, signal loss and so on. To explain the effective energy loss procedure after signal denoising, mean squared error (*MSE*) was used to explain the difference between the denoised signal and the original signal. The smaller the *MSE*, the smaller the signal loss, the better the signal reduction effect. NSR (Noise Suppression Ratio) is defined to reflect the noise suppression effect. The smaller NSR is, the better the denoising effect achieved^16^.

18 $$MSE=\sqrt{\frac{1}{N}\sum _{i=1}^{N}{({y}_{i}-{x}_{i})}^{2}}$$

19 $$NSR={[\frac{{\sum }_{t=1}^{T}{|f^{\prime} (t)-s(t)|}^{2}}{{\sum }_{t=1}^{T}{|f(t)-s(t)|}^{2}}]}^{\frac{1}{2}}$$

**Figure 10:**
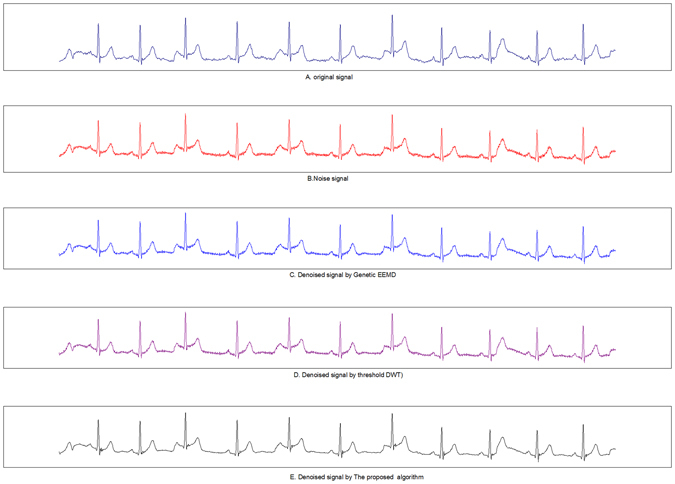
Comparison of denoising results with different denoising algorithms. A is the original ECG, B is the ECG containing noise, C is the ECG after processed by Threshold DWT, D is the ECG processed by Genetic EEMD, and E is the ECG processed by the algorithm in the paper.

From the experimental results shown in Table 3, the Proposed DWT algorithm is significantly superior to the other two representative denoising algorithms in terms of MSE, NSR and TIME. For the denoising effect, Proposed DWT has especially obvious advantages compared with Threshold DWT. For the CPU time consumption, the optimistic algorithms save half the time of Genetic EEMD. Figure 10 illustrates the comparison of the results after denoising of the first 10 seconds of data recorded by Person_01/rec_1.

**Table 3:** Comparison of the effect of three different denoising algorithms.

	Threshold DWT			Genetic EEMD			Proposed DWT		
	*MSE*	*NSR*	*TIME*(*us*)	*MSE*	*NSR*	*TIME*	*MSE*	*NSR*	*TIME*
Person_01/rec_1	0.116	0.101	48.2	0.087	0.073	78.1	0.067	0.051	37.9
Person_02/rec_1	0.117	0.102	48.1	0.077	0.064	78.2	0.067	0.057	37.5
Person_03/rec_1	0.116	0.101	48.3	0.097	0.082	78.2	0.087	0.069	38.6
Person_04/rec_1	0.117	0.103	48.2	0.077	0.073	78.1	0.087	0.065	39.6
Person_05/rec_1	0.115	0.102	48.1	0.087	0.089	78.3	0.077	0.073	37.4
Person_06/rec_1	0.116	0.101	48.2	0.077	0.079	78.2	0.087	0.065	39.7
Person_07/rec_1	0.117	0.101	48.3	0.077	0.068	78.1	0.067	0.059	37.9
Person_08/rec_1	0.116	0.103	48.2	0.097	0.097	78.1	0.077	0.082	36.4
Person_09/rec_1	0.115	0.101	48.3	0.077	0.085	78.3	0.067	0.078	37.6
Person_010/rec_1	0.116	0.103	48.1	0.097	0.077	78.2	0.077	0.069	38.5
average	0.1161	0.1018	48.2	0.085	0.0787	78.18	0.076	0.0668	38.11

## Conclusion

Although a classic method of processing an ECG signal is by using wavelet transform, there is still much confusion about ECG signal processing with wavelet. For example, on the premise that wavelet supporting width can be tolerated, is it true that the larger the order number of the vanishing moment, the more concentrated the energy generated when the signal is decomposed by the wavelet? How can we realize the precise filtering of ECG signal by wavelet transform and keep the singular point in the signal? In this paper, a quantitative analysis was performed to study the correlation between the vanishing moment and frequency characteristics of the wavelet, an optimal wavelet base function was selected based on the amplitude frequency characteristics of ECG signals, and wavelet bases of different orders were used to deal with different wavelet spaces. We also found that the accurate bandpass filtering could be realized in the process of wavelet transform according to the different frequency characteristics of noise, which effectively avoided the damage to the signal during the denoising process. Experimental results showed that the proposed wavelet transform with optimized parameters had a remarkable effect on ECG signal denoising and has a strong practical significance, including the ECG monitoring watch developed by myself.
